# Confusion in the interpretation of prolactin levels caused by inappropriately low reference intervals

**DOI:** 10.1530/EC-24-0432

**Published:** 2024-11-21

**Authors:** Yanaika S Sabogal Piñeros, Martine M L Deckers, Prim de Bie, Annemieke C Heijboer, Jacquelien J Hillebrand

**Affiliations:** 1 Endocrine Laboratory, Department of Laboratory Medicine, Amsterdam UMC location University of Amsterdam, Amsterdam, The Netherlands; 2 OLVG Lab, Department of Clinical Chemistry, Amsterdam, The Netherlands; 3 Endocrine Laboratory, Department of Laboratory Medicine, Amsterdam UMC location Vrije Universiteit Amsterdam, Amsterdam, The Netherlands; 4 Amsterdam Gastroenterology Endocrinology & Metabolism, Amsterdam, the Netherlands; 5 Amsterdam Reproduction & Development Research Institute, Amsterdam, The Netherlands

**Keywords:** immunoassay, normal values, standardization, method comparison, bias

## Abstract

**Objective:**

Serum prolactin measurements are important in the differential diagnosis of pituitary masses and subfertility. We observed discrepancies in serum prolactin levels in several patients measured with different immunoassays. Despite differences in assay results, the reference intervals (RIs) derived by the manufacturers were similar. In this study, we aimed to investigate prolactin assay differences and to re-establish RIs for different prolactin immunoassays.

**Methods:**

For the assay comparison, serum samples were collected from men and women visiting the Amsterdam UMC hospital. Prolactin levels were measured using the Atellica^TM^ IM analyzer (Siemens Healthineers) and the Cobas (Roche Diagnostics) immunoassay. RIs for prolactin were re-established for men, premenopausal women, and postmenopausal women for both the Atellica and Cobas immunoassay.

**Results:**

Prolactin levels measured using the Cobas immunoassay were 1.75 times higher than those measured using the Atellica immunoassay. The re-established RIs for Atellica and Cobas confirmed these findings and were <0.32 U/L; <0.55 U/L for men; <0.64 U/L; <0.86 U/L for premenopausal women, and <0.31 U/L; <0.59 U/L for postmenopausal women, respectively, for Atellica and Cobas assays. The re-established RIs of the Atellica assay matched the current and manufacturer RIs, whereas those for Cobas differed substantially.

**Conclusions:**

Prolactin levels are assay-dependent, and the re-established RIs are different for the Atellica and Cobas assays. We recommend that laboratory specialists and manufacturers periodically review prolactin assay RIs, as incorrect RIs can lead to misinterpretation of prolactin levels and unnecessary referrals and further laboratory testing, as we have experienced.

**Plain language summary:**

We showed that the results of different prolactin tests disagree by 75%, which hinders correct interpretation. Thus, we established test-specific prolactin normal values. By being aware of test differences and using test-specific normal values, one can ensure correct interpretation and prevent unnecessary referrals and concern.

## Introduction

Prolactin is produced in the anterior pituitary gland and plays a functional role in lactation and breast development (1, 2, 3). Elevated prolactin concentrations may be caused by prolactin-secreting pituitary adenomas, loss of dopamine inhibition by antipsychotic drugs, or systemic disorders (3, 4). Serum prolactin is a key laboratory test in the differential diagnosis of, among others, pituitary masses, infertility problems, and menstrual disorders. Serum prolactin is measured using immunometric immunoassays. Although most commercially available assays are standardized to the World Health Organization (WHO) 84/400 standard, there are still considerable standardization differences between assays from different manufacturers (5, 6, 7, 8, 9, 10, 11). Therefore, follow-up of prolactin levels should ideally be performed using the same assay and assay-specific reference intervals (RIs). Recently, we have encountered several cases of elevated (above the upper limit of normal (ULN) of the institutional RI) prolactin levels in women without clinical signs of prolactin excess, all measured with the Elecsys prolactin assay on the Cobas (Roche Diagnostics). Because of known standardization differences and because immunoassays, like prolactin assays, are susceptible to interferences, we re-analyzed these samples using a different immunoassay, the Atellica (Siemens Healthineers) prolactin assay. In all cases, prolactin levels were not elevated (<ULN of the institutional Atellica-based RI) when measured using the Atellica. Interestingly, both the institutional and the manufacturer’s suggested RI for the Atellica and Cobas assays were approximately similar, despite the bias between the two assays. The aim of this study is to gain more insight into prolactin assay differences and to establish institutional (assay-specific) prolactin RIs for both the Cobas and Atellica assays.

## Material and methods

This study was performed in the Endocrine Laboratory of the Amsterdam UMC (Lab 1, Prolactin assay on the Atellica^TM^ IM Analyzer (Siemens Healthineers)) and the OLVG Laboratory (Lab 2, Elecsys Prolactin II assay on Cobas e601 analyzer (Roche Diagnostics)). The institutional RIs for the Atellica and Cobas assays are <0.30 U/L, <0.37 U/L for men; <0.60 U/L and <0.62 U/L for premenopausal women; and <0.40 U/L and <0.43 U/L for postmenopausal women (Table 1). According to Dutch law, medical ethics committee approval is not required for measurements on residual sera to improve the quality of laboratory analyses. The local medical ethics committee confirmed this and stated that the Medical Research Involving Human Subjects Act does not apply to the collection of blood samples for improving the quality of laboratory analyses and determining RIs.

**Table 1:** Overview of prolactin reference intervals for men, premenopausal women, and postmenopausal women.

	Assay	Men	Premenopausal women	Postmenopausal women
**Current institutional RI for prolactin (U/L)**				
Amsterdam UMC	Atellica (Siemens)	<0.30	<0.60	<0.40
OLVG	Cobas (Roche)	<0.37	<0.62	<0.43
**Manufacturer’s RI for prolactin (U/L)**				
	Atellica (Siemens)	0.045–0.375	0.059–0.619	0.038–0.430
	Cobas (Roche)	0.086–0.324	0.102–0.496	Not mentioned
**Newly established RI for prolactin (U/L)**				
Amsterdam UMC	Atellica (Siemens)	<0.32	<0.64	<0.31
OLVG	Cobas (Roche)	<0.55	<0.86	<0.59

RI, reference interval. Institutional RIs, manufacturer RIs (from kit insert) and newly established RIs, for both Atellica (Siemens) and Cobas (Roche) and for men, premenopausal women, and postmenopausal women. RIs represent the 2.5th and 95th percentile values.

### Method comparison

Thirty random remnant serum samples from the outpatient clinic of Amsterdam UMC (10 men and 20 women, mean age of 46.4 years (range 17–77 years)) were collected. All remnant serum samples were stored at −20⁰C for less than 3 months and thawed only once. Prolactin was measured in split serum samples using the Atellica and Cobas assays according to the manufacturer’s protocol. Polyethylene glycol (PEG) precipitation (25% PEG 6000 in water) was used to screen for macroprolactin in samples with prolactin levels >1 U/L (Cobas assay). A recovery of monomeric prolactin of >60% was used as the cutoff to exclude macroprolactin.

### Establishment of reference intervals

RIs were established for healthy men, premenopausal women, and postmenopausal women using a direct approach. Two separate sets of RI samples were collected for the Atellica and Cobas because of insufficient sample volume (Table 2). Sample collection was performed using the same criteria. All groups consisted of at least 120 samples. We collected remnant lithium heparin plasma samples from patients who visited the Amsterdam UMC for blood withdrawal. We selected 617 remnant samples of thyroid-stimulating hormone (TSH) measurements from patients over 18 years of age (*n* = 193 men, *n* = 205 premenopausal women, *n* = 219 postmenopausal women), who were visiting the outpatient clinic of the Amsterdam UMC (mainly outpatients of the general internal medicine, vascular medicine, tropical medicine, and hematology departments, without suspicion of high prolactin levels) or were referred from general practice to the Amsterdam UMC for blood withdrawal only. All samples had TSH levels within the RI. Remnant samples were stored at −20⁰C for less than 3 months and thawed only once. In addition, we used 126 serum samples of healthy employees that were collected for a RI study of thyroid hormones (*n* = 55 men, *n* = 41 premenopausal women, *n* = 30 postmenopausal women) (12). Written consent was obtained from all participants. Prolactin levels in samples from the healthy employees were not different from prolactin levels of the patients (independent *t*-tests); therefore, samples were combined for analyses.

**Table 2:** Descriptive statistics of the three defined groups and the reference intervals.

Assay	Group	*n*	Age (range) (yrs)	RI (U/L)	90% CI of the lower limit	90% CI of the upper limit
Atellica (Siemens)	Men	126	48 (20–89)	<0.32	0.06–0.07	0.27–0.54
	Premenopausal women	120	35 (18–50)	<0.64	0.05–0.08	0.45–1.50
	Postmenopausal women	120	63 (51–87)	<0.31	0.05–0.07	0.24–0.34
Cobas (Roche)	Men	121	51 (18–90)	<0.55	0.04–0.09	0.39–0.62
	Premenopausal women	120	35 (18–50)	<0.86	0.08–0.11	0.77–0.98
	Postmenopausal women	120	63 (51–87)	<0.59	0.07–0.09	0.47–1.08

RI, reference interval. RIs are provided with a 90% CI specified for the Atellica (Siemens) and Cobas (Roche) assay.

After measuring prolactin, the medical records of patients with prolactin concentrations >0.8 U/L (*n* = 14) were checked. Based on this review, we excluded samples from patients who were using atypical antipsychotics (*n* = 2), opioids (*n* = 1), or who were pregnant (*n* = 4). The remaining samples with high prolactin levels (*n* = 7) were not excluded. In the absence of clinical information on the menstrual cycle, the criteria for the postmenopausal state in women were set at age >50 years and serum follicle-stimulating hormone (FSH) >30 U/L (measured using Alinity (Abbott Diagnostics) or Cobas e601 (Roche Diagnostics)) (13, 14). After measuring FSH, nine samples of women over 50 years of age with FSH ≤30 were excluded.

### Statistical analysis

Passing and Bablok regression analysis and the establishment of the RIs were performed using MedCalc® (version 22.016, Oostende, Belgium). The Spearman correlation coefficient was calculated between both assays. The RIs were determined using a non-parametric percentile method (CLSI C28-A3). Independent *t*-tests were used to test whether prolactin levels were different between healthy volunteers and patients. Graphs were made with GraphPad Prism® version 5.0.

## Results

### Method comparison

**Figure 1:**
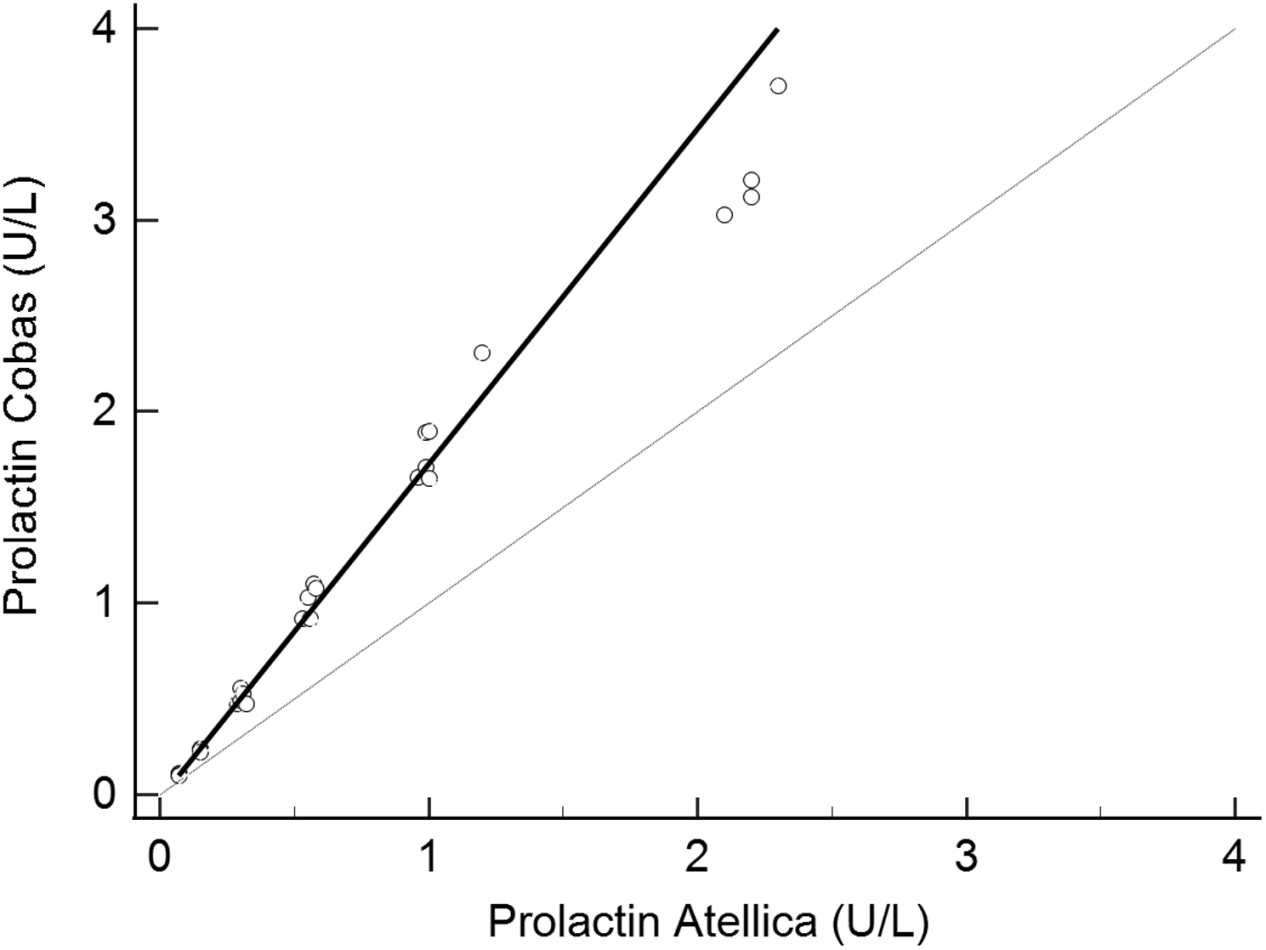
Passing–Bablok regression analysis for the Atellica and Cobas prolactin assay. Solid line: regression line, *y* (Cobas) = −0.0225 + 1.750× (Atellica). Dashed line: x = y line.

Thirty samples were included in the method comparison, with prolactin concentrations ranging from 0.07 to 2.30 U/L (Atellica). Passing–Bablok regression showed Prolactin_Cobas_ = −0.02 + 1.75× Prolactin_Atellica_ (95% CI 1.61–1.93), with a Spearman correlation coefficient of 0.986 (Fig. 1). Hence, prolactin levels were 1.75× higher when measured using the Cobas assay compared to the Atellica assay. When comparing only samples with serum prolactin levels within the institutional RI (Atellica, 0.07–0.58 U/L, *n* = 20), the bias between the Atellica and Cobas immunoassays increased to 85% (Prolactin_Cobas_ = −0.04 + 1.85 prolactin_Atellica_.

### Establishment of reference intervals

We collected plasma from 366 individuals to determine the RIs for the Atellica prolactin assay and from 361 individuals to determine the RIs for the Cobas prolactin assay. The newly established Atellica RIs were <0.32 U/L for men, <0.64 U/L for premenopausal women, and <0.31 U/L for postmenopausal women. The newly established Cobas RIs were <0.55 U/L for men, <0.86 U/L for premenopausal women, and <0.59 U/L for postmenopausal women.

## Discussion

We showed a significant difference in prolactin levels measured using the Atellica and Cobas assays. Prolactin concentrations measured with the Cobas assay were significantly higher, despite the use of similar RIs in clinical practice.

In the absence of a reference method, current prolactin immunoassays suffer from standardization differences, meaning that measured concentrations may differ between different immunoassays. In these circumstances, the use of method-dependent RIs is essential, as misinterpretation of prolactin levels may have major clinical implications. Although manufacturers of prolactin assays provide RIs, laboratories should preferably establish their own RIs for their population (requiring many samples, often also from different age groups and sexes) or verify (with considerably fewer samples) whether the proposed RIs are adequate for their patient population.

We performed a method comparison study and re-established prolactin RIs by a direct method for both the Atellica and Cobas assays. The re-established Atellica prolactin RIs confirmed the appropriateness of the current Atellica institutional RIs and manufacturer RIs for men and premenopausal women. The new RI for postmenopausal women was lower (current RI <0.40 U/L, new RI <0.31 U/L). This may be explained by a more homogeneous sample set than previously, as all women were selected based on age and FSH levels. However, the re-established Cobas prolactin RIs were different from the currently used institutional RIs for all groups studied. The RIs provided by the manufacturer were even more inappropriate (Table 1). The Cobas prolactin immunoassay is frequently used in the Netherlands and worldwide. Using the provided manufacturer’s RIs may lead to unnecessary referrals and incorrect medical decisions for many patients.

Our results are consistent with those of others showing that higher prolactin levels generated by the Cobas prolactin assay do not translate into higher manufacturer RIs. Ilardo et al. (6) showed, by using an indirect (retrospective) method, that Cobas prolactin levels were age- and sex-dependent and that the manufacturer’s RIs differed from their re-established prolactin RIs. In an Australian method comparison study, higher prolactin levels were found when using the Cobas assay compared to the Siemens Centaur assay (using the same reagents as the Atellica assay) (7). The authors subsequently evaluated the clinical history of 18 patients, confirming that the Cobas assay overestimated prolactin levels rather than the Centaur assay underestimating prolactin levels. The authors suggested that the inter-assay discordance in prolactin levels could be overcome by using higher Cobas RIs (7). Finally, a study from the United States established direct RIs for men and premenopausal women with and without hormonal contraception using the Cobas and Atellica prolactin assays (5). They found that both the established and the manufacturer’s RIs for the Atellica assay were consistent, but the established RIs for Cobas were considerably higher than the RIs provided by Roche. In contrast to our study, they did not establish RIs for postmenopausal women. These studies highlight both the robustness of our findings, given the various geographical regions, and the notable clinical implications. All studies, including ours, suggested that the prolactin overestimation by the Cobas assay could lead to unnecessary patient referrals, laboratory analyses, imaging, or treatment. Unfortunately, despite these findings, Roche has not yet re-established the RIs of their prolactin assay.

The strength of our study is that we showed assay differences between the Atellica and the Cobas assays and newly established RIs for men, premenopausal women, and postmenopausal women for both immunoassays. The differences we observed in the method comparison study were corroborated by the new RIs. The direct RI approach (using at least 120 considered healthy individuals per group according to CLSI guidelines (15)) was performed in all clinically relevant age and sex groups. Postmenopausal status was confirmed by an elevated plasma FSH concentration. Although the two separate sample sets may have introduced a bias, we believe this is not likely, as the samples were carefully selected according to the same criteria, and the factorial difference found in the RIs is consistent with the results of the method comparison.

Unexpected prolactin levels in individual cases may also be caused by analytical interferences, like macroprolactin or heterophilic antibodies. We have no indication that the method difference observed is based on random analytical interference, as the presence of macroprolactin was excluded using polyethylene glycol precipitation (data not shown).

In conclusion, our study shows a significant difference between two prolactin immunoassays (Cobas and Atellica) and highlights the importance of establishing or at least verifying prolactin RIs in laboratories when implementing a prolactin immunoassay. Furthermore, it is important to review RIs in a timely manner, as assay drift can occur, resulting in improper RIs over time. This applies to both laboratories and manufacturers. Inappropriate prolactin RIs may lead to the misinterpretation of prolactin levels, unnecessary referrals, additional diagnostics, and possibly incorrect diagnosis and treatment.

## Declaration of interest

There is no conflict of interest that could be perceived as prejudicing the impartiality of the research reported.

## Funding

This research did not receive any specific grant from any funding agency in the public, commercial, or not-for-profit sector.
